# O_2_ evolution and recovery of the water-oxidizing enzyme

**DOI:** 10.1038/s41467-018-03545-w

**Published:** 2018-03-28

**Authors:** Keisuke Kawashima, Tomohiro Takaoka, Hiroki Kimura, Keisuke Saito, Hiroshi Ishikita

**Affiliations:** 1 Department of Applied Chemistry, The University of Tokyo, 7-3-1 Hongo, Bunkyo-ku, Tokyo, 113-8654 Japan; 2 Research Center for Advanced Science and Technology, The University of Tokyo, 4-6-1 Komaba, Meguro-ku, Tokyo, 153-8904 Japan

## Abstract

In photosystem II, light-induced water oxidation occurs at the Mn_4_CaO_5_ cluster. Here we demonstrate proton releases, dioxygen formation, and substrate water incorporation in response to Mn_4_CaO_5_ oxidation in the protein environment, using a quantum mechanical/molecular mechanical approach and molecular dynamics simulations. In S_2_, H_2_O at the W1 site forms a low-barrier H-bond with D1-Asp61. In the S_2_-to-S_3_ transition, oxidation of O_W1_H^–^ to O_W1_^•–^, concerted proton transfer from O_W1_H^–^ to D1-Asp61, and binding of a water molecule W_*n*-W1_ at O_W1_^•–^ are observed. In S_4_, W_*n*__-W1_ facilitates oxo-oxyl radical coupling between O_W1_^•–^ and corner μ-oxo O4. Deprotonation via D1-Asp61 leads to formation of O_W1_=O4. As O_W1_=O4 moves away from Mn, H_2_O at W539 is incorporated into the vacant O4 site of the O_2_-evolved Mn_4_CaO_4_ cluster, forming a μ-oxo bridge (Mn3–O_W539_–Mn4) in an exergonic process. Simultaneously, W_*n*-W1_ is incorporated as W1, recovering the Mn_4_CaO_5_ cluster.

## Abstract (web summary)

Water splitting during photosynthesis results in the combination of two oxygen atoms to form O_2_. Here, based on computational simulations, the authors develop a possible mechanism for this reaction, which is different from the mechanisms previous studies have suggested.

## Introduction

**Fig. 1:**
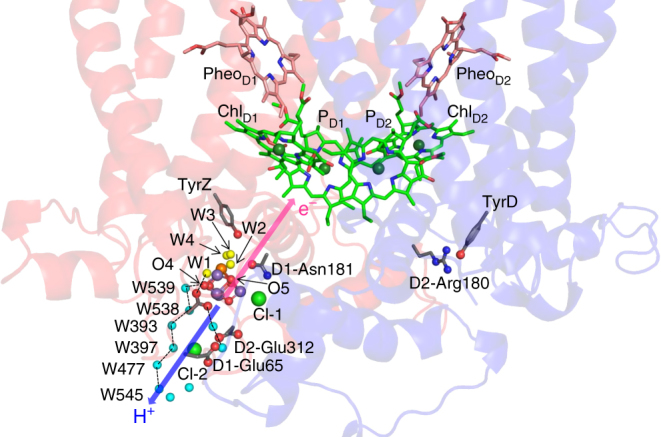
Proton and electron transfer pathways near Mn_4_CaO_5_. Blue and pink arrows indicate proton and electron transfer pathways proceeding from the Mn_4_CaO_5_ cluster, respectively

Dioxygen can be produced by removing four electrons and four protons from two substrate water molecules: 2H_2_O → O_2_ + 4 H^+^ + 4e^–^. In a protein-pigment complex photosystem II (PSII), the water-oxidation reaction proceeds at the oxygen-evolving complex via the S_*n*_-state transitions (*n* represents the total number of oxidation steps)^1–3^. The oxygen-evolving complex is linked with electron transfer pathways, proton transfer pathways, and substrate water intake pathways. Excitation of P680, which is composed of four chlorophyll *a* molecules, leads to electron transfer to the second plastoquinone, Q_B_, via pheophytin *a* and the first plastoquinone, Q_A_. The resulting P680^•+^ state is stabilized by electron transfer from the Mn_4_CaO_5_ cluster via redox active D1-Tyr161 (TyrZ) (Fig. 1).

Protons released from substrate water molecules are transferred via proton transfer pathways (e.g., O4-water chain^4^), which proceeds from the Mn_4_CaO_5_ cluster toward the protein bulk surface (Fig. 1). The releases of protons are observed with a typical stoichiometry of 1:0:1:2 for the S_0_ → S_1_ → S_2_ → S_3_ → S_0_ transitions, respectively^5^. The O4-water chain is a chain of water molecules, which forms an H-bond with the O4 site of the Mn_4_CaO_5_ cluster via a water molecule W539^4^. Since the rate constant is pH-independent in the S_0_-to-S_1_ transition, the absence of ionizable groups in the O4-water chain fits with the suggestion that the O4-water chain is the relevant proton transfer pathway^5,6^. Although release of a proton is not observed in the S_1_-to-S_2_ transition, it seems likely that migration of a proton or internal proton transfer can occur (e.g., ref. ^7^). On the other hand, the rate constant for the S_2_-to-S_3_ transition is pH-dependent, which suggests that ionizable groups are involved in the proton-transfer pathway^5,8^. Ionizable groups that are not ligands near the Mn_4_CaO_5_ cluster are D1-Asp61 and CP43-Arg357^9^. In the S_3_-to-S_0_ transition, O–O bond formation and release of O=O occur. The rate constant of 1–2 ms involved in the S_3_-to-S_0_ transition is pH-independent with a kinetic isotope effect (*k*_D_/*k*_H_) of 1.4^6^, and may correspond to O_2_ release and incorporation of a substrate water molecule^6,10^. *k*_D_/*k*_H_ = 1.4 is also typical for alteration in the H-bond pattern along water chains before and after proton transfer (pre-PT and post-PT H-bond patterns, respectively) in the Grotthuss mechanism^11^. Once proton transfer occurs, the pre-PT pattern transforms to the post-PT pattern^4^.

The slow-exchanging and fast-exchanging water molecules are candidate substrates in the Mn_4_CaO_5_ cluster^12^. In the S_0_-to-S_1_ transition, the exchange rate of the slow-exchanging water molecule decreases 600 times^12^ and deprotonation of the slow-exchanging water molecule may occur, indicating that it may be a μ-OH bridge of the Mn_4_CaO_5_ cluster in S_0_^13^. In the S_1_-to-S_2_ transition, the exchange rate of the slow-exchanging water molecule increases 100 times^12^. The exchange rate of the fast-exchanging water molecule decreases three times in the S_2_-to-S_3_ transition^14^. The fast-exchanging water molecule may be a terminal ligand to Mn[2] and possibly form an H-bond with D1-Asp61^13,15^. The corresponding water molecule is W1 in the crystal structure analyzed at 1.9 Å resolution^9^. These water molecules approach the catalytic site from the bulk region via water channels. The D1-Glu65/D2-Glu312 channel is a candidate substrate water intake channel. The D1-Glu65/D2-Glu312 channel provides water molecules near TyrZ and the Mn_4_CaO_5_ cluster. It can also deliver water molecules to the W539 and W538 sites in the O4-water chain via D1-Asp61^16^.

Recently, the two-flash illuminated PSII structure analyzed at 2.35 Å resolution was reported, demonstrating several characteristic O sites in the Mn_4_CaO_6_ moiety^17^ (e.g., W538, W539, O4, and O6). W538^9^ (i.e., W665^17^) was unambiguously identified in both the 1.9 Å^9^ and radiation-damage-free^18^ PSII crystal structures^4^, whereas it was absent in the two-flash illuminated PSII structure. W538 forms an H-bond with W539. The averaged O4…O_W539_ (i.e., O4…O_W567_^17^) distance of 2.32 Å identified in the two-flash illuminated PSII structure is exceptionally short in comparison with the typical O–O distances of ~2.8 Å for standard (asymmetric) H-bonds in H_2_O. The two-flash illuminated PSII structure shows the presence of O6 near O5. The O6…O5 distance is 1.5 Å, which is close to the distance for peroxide (O–O)^2–^. This suggests that formation of O=O may occur at this site, which fits the reaction model proposed by Siegbahn et al.^19^. On the other hand, it is unclear whether the reaction model could also explain how the other characteristic O sites (e.g., W538, W539, and O4) are involved in the mechanism of water oxidation. It should be noted that the *B*-factor values of these O atoms are at the same level in the two-flash illuminated PSII structure.

Here we analyzed alterations in the Mn_4_CaO_5_ structure and H-bond/water network in response to Mn_4_CaO_5_ oxidation in the PSII protein environment, using a quantum mechanical/molecular mechanical (QM/MM) approach and molecular dynamics (MD) simulations based on the PSII crystal structure. In S_2_, the proton of H_2_O at W1 significantly migrates toward D1-Asp61 due to oxidation of Mn4, forming a low-barrier H-bond (HO_W1_^–^…H^+^…^–^OOC_D1-Asp61_). In the S_2_-to-S_3_ transition, oxidation of O_W1_H^–^ to O_W1_^•–^ preferentially occurs with respect to oxidation of Mn1(III) to Mn1(IV), because D1-Asp61 accepts the proton from O_W1_H^–^ and facilitates oxidation to O_W1_^•–^ via proton-coupled electron transfer (i.e., decreasing the redox potential of W1 for oxidation). In S_3_, a water molecule binds at O_W1_^•–^. Incorporation of water molecules (including W2 and W3) into the open-cubane O5 site are not observed. Since the nearest μ-oxo to O_W1_^•–^ is O4, oxo-oxyl radical coupling occurs between O4 and O_W1_^•–^ in S_4_, resulting in (O_W1_–O4)^2–^ formation. As O_W1_=O4 moves away from the Mn3/Mn4 moiety, H_2_O at W539 is incorporated into the vacant O4 site near CP43-Arg357, forming a μ-oxo bridge with Mn3–O_W539_ and Mn4–O_W539_ in a concerted exergonic process. Incorporation of W539 into the O4 site requires reorientation of W539, which induces alterations in the H-bond pattern from the post-PT pattern to pre-PT pattern along the O4-water chain. A water molecule, which binds at O_W1_^•–^ in S_3_, is also incorporated into the vacant W1 site (i.e., new W1), recovering the Mn_4_CaO_5_ cluster.

## Results

### Formation of a low-barrier H-bond between W1 and D1-Asp61

**Fig. 2:**
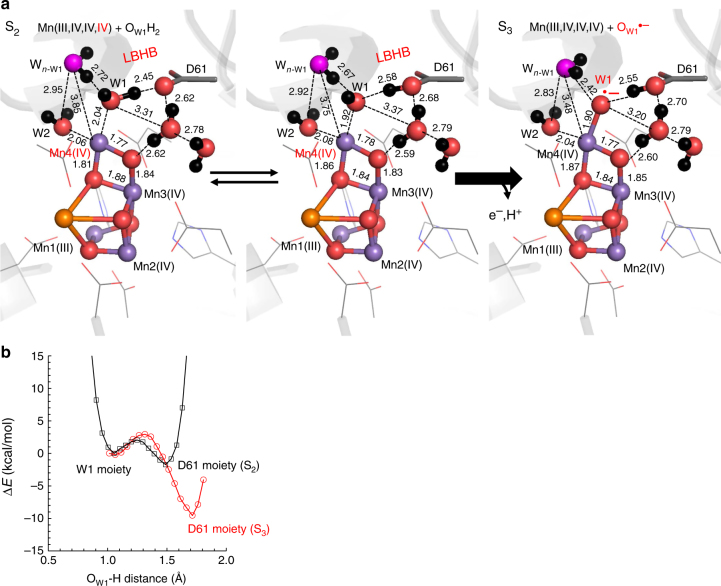
The open-cubane S_2_-to-S_3_ transition. **a** QM/MM geometries. Mn, Ca, O, and H atoms are represented by purple, orange, red, and black balls, respectively. Dotted lines indicate distances (excluding H atoms). **b** The energy profiles of the H-bonds between W1 (H_2_O/OH^–^) and D1-Asp61 in S_2_ and W1(OH^•^/O^•–^) and D1-Asp61 in S_3_. The black curve corresponds to release of the proton from H_2_O at W1 to D1-Asp61 in S_2_, whereas the red curve corresponds to release of the proton from OH^•^ at W1 to D1-Asp61 in S_3_. The spin configurations (e.g., high, low, ferromagnetic, and antiferromagnetic) did not essentially affect the resulting optimized Mn_4_CaO_5_ geometries and potential-energy profiles (Table 1 and Supplementary Figure 1)

We used the deprotonated open-cubane S_2_ structure, where (Mn1,Mn2,Mn3,Mn4) = Mn(III,IV,IV,IV), as it seems to be energetically more stable than the closed-cubane S_2_ structure^20–23^. The p*K*_a_ of H-bond donor and acceptor moieties in H-bonds can be analyzed from the potential-energy profiles of the H-bonds^24–26^. In H-bonds, a proton is more likely to populate the moiety with the higher p*K*_a_ value between the two moieties^26^. The energy difference between the H-bond donor and acceptor moieties corresponds to the p*K*_a_ difference and this feature also holds true for H-bonds in protein environments^27^. QM/MM calculations showed that a short H-bond between H_2_O at W1 and D1-Asp61 (O_W1_…O_D1-Asp61_ = 2.45 Å) was formed in the open-cubane S_2_ (Fig. 2a). The potential-energy profile suggested that the O_W1_…O_D1-Asp61_ H-bond is a low-barrier H-bond (LBHB, where the p*K*_a_ difference for the donor and acceptor moieties is nearly zero^25,27^) (Fig. 2b), and that the proton is significantly migrated toward D1-Asp61 and delocalized over the two moieties already in S_2_ (Fig. 2a). This may correspond to the significant changes in the H-bond properties between D1-Asp61 and a water molecule in the S_1_-to-S_2_ transition observed in Fourier transform infrared (FTIR) spectroscopy^7^. It should also be noted that the spin configurations (e.g., high, low, ferromagnetic, and antiferromagnetic) did not essentially affect the resulting optimized Mn_4_CaO_5_ geometry and energies in the present case (Table 1). Potential-energy profiles also remained unaffected since proton transfer does not involve oxidation of Mn ions (Supplementary Figure 1).

**Table 1:** Spin configurations and the resulting optimized Mn_4_CaO_5_ geometries (in Å) and energies (in kcal/mol) calculated for antiferromagnetically coupled Mn ions with respect to ferromagnetically coupled Mn ions in S_2_

	Total spin *S*	RMSD (Å)	Δ*E* (kcal/mol)
Antiferromagnetic	1/2	0.007	−1.7
Antiferromagnetic	5/2	0.007	−1.1
Antiferromagnetic	7/2	0.007	−0.6
Ferromagnetic	13/2	—	—

RMSD root-mean-square deviation, — not applicable

### Absence of Mn oxidation in the S_2_-to-S_3_ transition

Unexpectedly, oxidation of S_2_ to S_3_ did not involve oxidation of Mn; however, oxidation of O_W1_H^–^ to O_W1_^•–^ and concerted proton transfer from O_W1_H^–^ to D1-Asp61 did occur, resulting in Mn(III,IV,IV,IV)…O_W1_^•–^…HOOC_D1-Asp61_ in the open-cubane conformation (Fig. 2a). Accordingly, the first proton of W1 (from H_2_O/OH^–^), which was delocalized over the low-barrier O_W1_…H^+^…O_D1-Asp61_ bond in S_2_, seems likely to have been released toward the bulk surface via D1-Asp61 (along the D1-Glu65/D2-Glu312 water channel) in the S_2_-to-S_3_ transition^16,28^, since the potential-energy profile indicated that even the second proton from W1 (from OH^•^/O^•–^) can be released to D1-Asp61 (Fig. 2). MD simulations indicated that D1-Asp61 was able to accept a proton by rotating the protonated O site (Supplementary Figure 2), as reported in QM/MM-MD simulations by Narzi et al.^29^. Since p*K*_a_(OH^–^/ O^2–^) ( = 24) > p*K*_a_(OH^•^/ O^•–^) (=12^30^), the concerted oxidation and proton release of O_W1_H^–^ to O_W1_^•–^ occurs without stabilizing O_W1_H^•^ on Mn4(IV).

In the S_2_-to-S_3_ transition, it remains unclear whether a Mn-centered oxidation (e.g., ref. ^31^) or a ligand-centered oxidation (e.g., ref. ^32^) occurs (discussed in refs. ^5,13,33,34^). Some theoretical studies favor the Mn-centered oxidation model over the ligand-centered oxidation model (e.g., refs. ^35–39^). Recent electrochemical analysis using the α-MnO_2_ electrode shows that addition of carboxylic acid (benzoic acid) induced proton-coupled electron transfer, resulting in a decrease in the redox potential required for water oxidation and an increase in evolved O_2_ at lower potentials^40^. Remarkably, FTIR spectra suggest that benzoic acids do not directly ligate to the Mn ion but form an H-bond with a ligand water molecule of the Mn ion, as identified in the Mn4…W1…D1-Asp61 moiety in PSII. The presence of benzoic acid as the proton acceptor facilitates proton-coupled electron transfer and can avoid accumulation of the positive charge in response to oxidation of Mn, leading to a decrease in the redox potential required for oxidation of water^40^. This may hold true for the present case, where proton-coupled electron transfer from W1 to D1-Asp61 occurs in response to the S_2_-to-S_3_ transition, which can specifically decrease the redox potential of W1 for one-electron oxidation.

The absence of the Mn-centered oxidation in the S_2_-to-S_3_ transition has been reported in spectroscopic studies by Messinger et al.; Mn was not oxidized, but a ligand substrate molecule was oxidized in the S_2_-to-S_3_ transition and an O radical was present in S_3_ (Mn(III)Mn(IV)_3_)^32^, which is consistent with the present results. It should be noted that although TyrZ and the H-bond network were also considered quantum-chemically (i.e., in the QM region), we did not observe formation of TyrZ^•^.

**Fig. 3:**
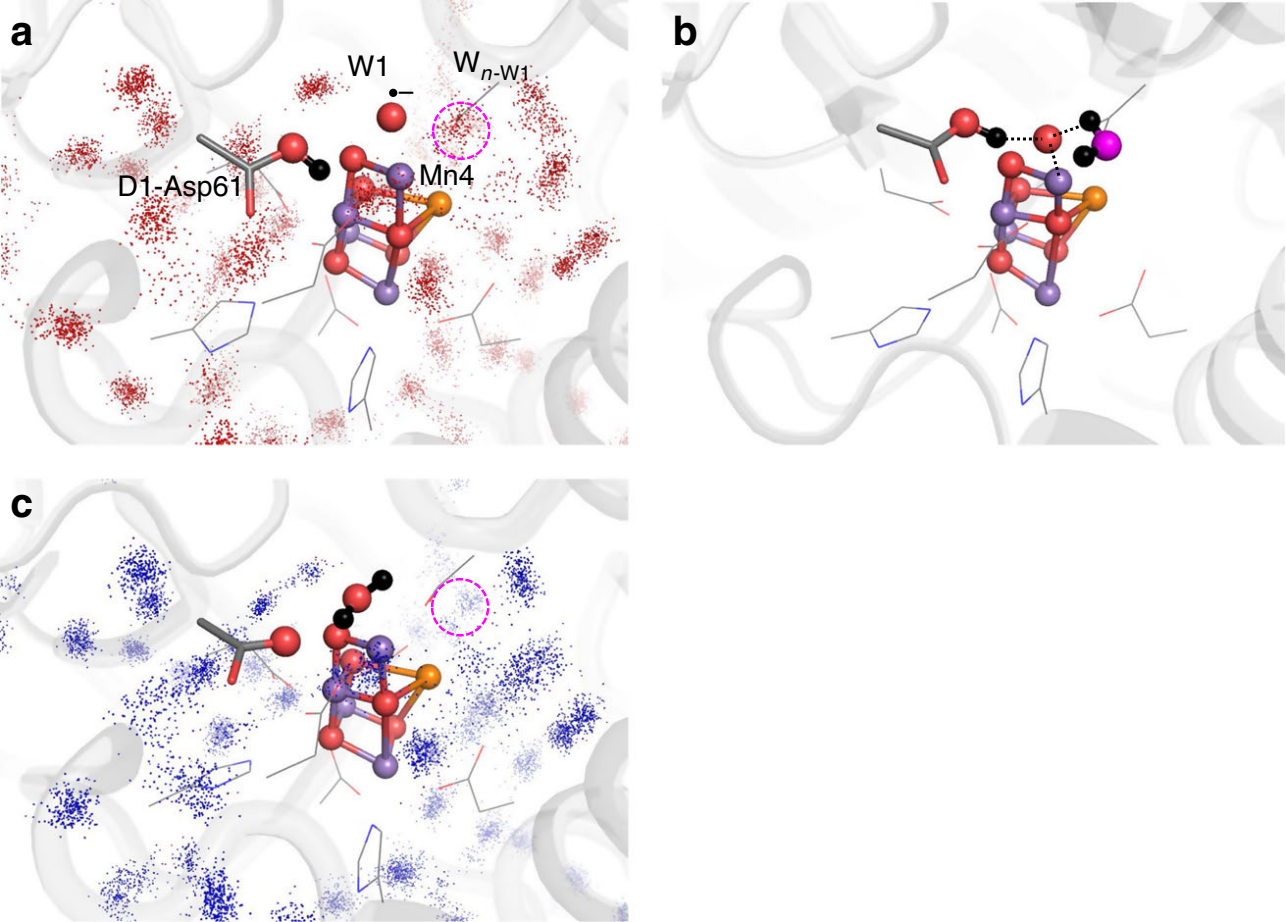
Water distribution near the Mn_4_CaO_5_ cluster. **a** Water distribution in S_3_ after an equilibrating MD run at 45−50 ns (250 snapshots). The position of W_*n*__-W1_ is indicated by the dotted circle. **b** The corresponding view in the QM/MM-optimized geometry in S_3_. Dotted black lines indicate interactions. **c** Water distribution in S_1_ after an equilibrating MD run at 45−50 ns (250 snapshots). W_*n*__-W1_ is absent in S_1_  (dotted circle)

According to the interpretation of FTIR difference spectroscopy, a water molecule was incorporated into the Mn_4_CaO_5_ moiety in the S_2_ to S_3_ transition^41^. In S_3_, MD simulations showed that a water molecule existed near W1 (W_*n*__-W1_) and could form an H-bond with O_W1_^•–^ (Fig. 3). The corresponding water molecule is absent in the PSII crystal structures^9,18^. The binding of W_*n*__-W1_ seems to be made more pronounced by the negatively charged O_W1_^•–^ in S_3_ (Supplementary Table 1). Near W_*n*__-W1_ there exists D1-Ser169, which has been proposed to provide access to water molecules for the substrate-binding site^42^. QM/MM calculations also showed that the H-bond between W1 and W_*n*__-W1_ is remarkably short: 2.42 Å in S_3_ (i.e., O^•–^ at W1; Fig. 2a) compared with 2.72 Å in S_2_ (i.e., H_2_O at W1; Fig. 2a). MD simulations suggested that water molecules at the W_*n*__-W1_ binding moiety are exchangeable with bulk water via the D1-Glu65/D2-Glu312 water channel^16^, indicating that the D1-Glu65/D2-Glu312 water channel can serve as the intake channel for W_*n*__-W1_.

We did not observe any incorporation of water molecules (including W2 and W3) into the open-cubane O5 site, nor did we observe formation of O_W2_^•–^ in QM/MM calculations, because of the absence of the proton acceptor groups (e.g., D1-Asp61). Below, we focus on the Mn_4_CaO_5_ cluster based on the obtained open-cubane S_3_ with O_W1_^•–^.

### Oxidation of S_3_ and formation of (O_W1_–O4)^2–^

**Fig. 4:**
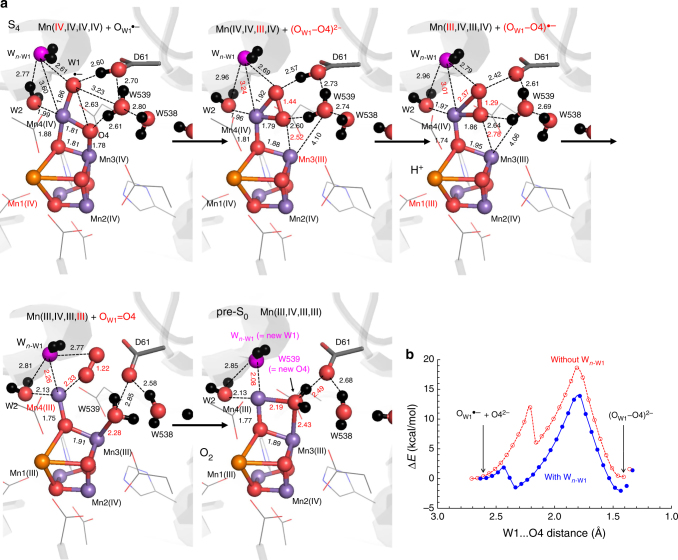
The S_4_ and pre-S_0_ states. **a** QM/MM geometries. Mn, Ca, O, and H atoms are represented by purple, orange, red, and black balls, respectively. Dotted lines indicate distances (excluding H atoms). Release of (O_W1_–O4)^•–^ from the Mn3/Mn4 moiety (i.e., the Mn3…O4 distance was increased) resulted in formation of O_W1_=O4. See supplementary Figure 4 for the energy profile. **b** The energy profiles of the (O_W1_–O4)^2^^−^ formation (the first and second panels in **a**) in the presence (blue curve) and absence (red curve) of W_*n*__-W1_. The initial lower barriers correspond to reorientation of the carboxylate group of D1-Asp61 with respect to O_W1_^•–^/(O_W1_–O4)^2–^

Oxidation of S_3_ resulted in Mn(IV,IV,IV,IV)…O_W1_^•–^…HOOC_Asp61_ (we denote the oxidized S_3_ states as “S_4_”, Fig. 4a). The presence of O_W1_^•–^ in S_4_ suggests that W1 may be involved in O–O single-bond formation via oxo-oxyl radical coupling. The nearest μ-oxo to O_W1_^•–^ is O4. Since we observed neither formation of O_W2_^•–^ nor a reactive Mn(V)=O species, we focused on O_W1_^•–^ and O4.

The potential-energy profile suggests that as oxo-oxyl radical coupling (e.g., ref. ^43^) proceeds between O4 and O_W1_^•–^, formation of (O_W1_–O4)^2–^ (confirmed by O_W1_–O4 = 1.44 Å and the spin density (2*S*) = 0 for both O_W1_ and O4) and concerted electron transfer from the (O_W1_–O4) moiety to Mn3 occur, resulting in Mn(IV,IV,III,IV)…(O_W1_–O4)^2–^…HOOC_Asp61_ (Fig. 4a). The energy barrier for the (O_W1_–O4)^2–^ formation was 13.9 kcal/mol (Fig. 4b), which is essentially consistent with the estimated values (e.g., 12.1 kcal/mol^44^). In all S-state transitions, including the (O_W1_–O4)^2–^ formation process, Mn2(IV) is not involved in oxidation/reduction, which could explain the similar energy barriers in the antiferromagnetically (↑↓↑↑) (Fig. 4) and ferromagnetically (↑↑↑↑) (Supplementary Figure 3) coupled forms. The energy barrier was 18.6 kcal/mol in the absence of W_*n*__-W1_ (Fig. 4b and Supplementary Figure 3). Because W_*n*__-W1_ lowers the energy barrier, it may serve as a catalyst for (O_W1_–O4)^2–^ formation in S_4_. As formation of (O_W1_–O4)^2–^ proceeded, the H atom of W_*n*__-W1_, which was initially oriented toward O_W1_^•–^, became oriented away from (O_W1_–O4)^2–^. Simultaneously, the W_*n*__-W1_…Mn4 distance decreased from 3.60 to 3.24 Å, owing to displacement of W1 toward O4, allowing W_*n*__-W1_ to approach Mn4 (Fig. 4).

### Formation of O_W1_=O4

When D1-Asp61 was deprotonated in the presence of (O_W1_–O4)^2–^, QM/MM calculations resulted in formation of (O_W1_–O4)^•–^ (e.g., ref. ^45^, confirmed by O_W1_–O4 = 1.29 Å and the spin density (2*S*) = 0.8 for O_W1_ and 0.4 for O4) and concerted electron transfer to Mn1 (Fig. 4).

Furthermore, the following events occurred concertedly: (i) W_*n*__-W1_ approached Mn4, (ii) (O_W1_–O4)^•–^ moved away from Mn3/Mn4, (iii) W539 approached Mn3, and (iv) electron transfer from (O_W1_–O4)^•–^ to Mn4 occurred, resulting in formation of O_W1_=O4 (confirmed by O_W1_–O4 = 1.22 Å and the spin density (2*S*) = 1.0 for O_W1_ and 0.9 for O4; Fig. 4) in an exergonic process (Supplementary Figure 4). The resulting oxidation state was Mn(III,IV,III,III), returning to an Mn oxidation state identical to that of S_0_[4].

After O_2_ evolution, the O_2_-evolved Mn_4_Ca“O_4_” cluster formed, which resembled the O4-lacking Mn_4_CaO_4_ cluster reported in the 3.5-Å PSII crystal structure^46^ and the O4-lacking synthetic Mn_4_CaO_4_ cluster reported by Zhang et al.^47^. The evolved O_2_ molecule is present in the hydrophobic space between Mn4 and D1-Ile60 (next to D1-Asp61), which has been proposed to serve as an O_2_-exiting pathway^48^. Indeed, O_2_ evolution was significantly lowered when D1-Ile60 was mutated to bulky phenylalanine^49^.

### Release of O_2_ and incorporation of W539 into the O4 site

Intriguingly, when evolved O_2_ was absent near Mn3/Mn4, QM/MM calculations resulted in exergonic incorporation of H_2_O at W539 (in the O4-water chain^4^, Fig. 1) into the vacant O4 site of the Mn_4_CaO_4_ cluster and formation of a μ-oxo bridge with Mn3–O_W539_ (2.43 Å) and Mn4–O_W539_ (2.19 Å) (pre-S_0_ state, Fig. 4). While W539 was incorporated into the Mn_4_CaO_4_ cluster, W_*n*__-W1_ simultaneously approached Mn4 and was incorporated into the Mn_4_CaO_5_ cluster as the new W1 ligand (W_*n*__-W1_…Mn4 = 2.08 Å). The resulting QM/MM geometry (Fig. 4) was quite similar to that of the original PSII crystal structure, demonstrating the ability to recover the initial state in the Kok cycle.

**Fig. 5:**
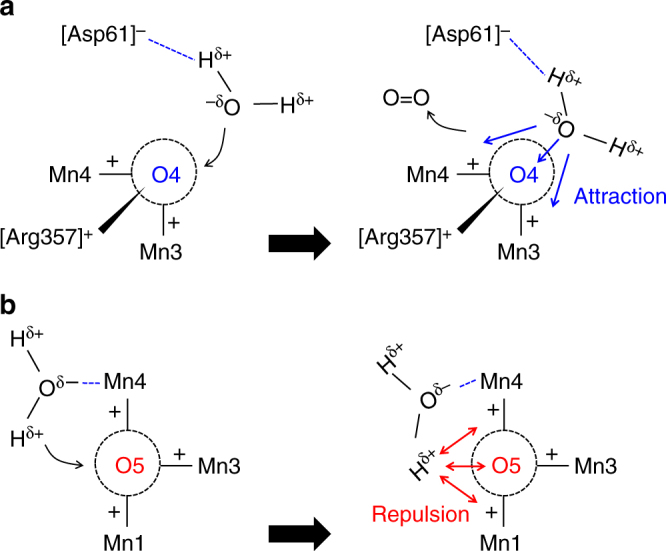
Difference in the orientations and interactions of the incorporating H_2_O molecule. **a** The O4 site. Anionic D1-Asp61 attracts the H^δ+^ and orients the O^δ–^ of the incorporating H_2_O toward cationic Mn3(III), Mn4(III), and CP43-Arg357, resulting in attraction. **b** The O5 site. The absence of an anionic H-bond acceptor and the nature of the incorporating H_2_O as a ligand of Mn4 force the H^δ+^ of the incorporating H_2_O to face cationic Mn1(III), Mn3(III), and Mn4(III), resulting in repulsion. The absence of D1-Asp61 near O5 would be energetically disadvantageous for incorporating H_2_O into the O5 site. O4 is located at the “corner” of the Mn3–O4 and O4–Mn4 bonds. Due to the presence of D1-Asp61, O_W539_^δ–^ can always be oriented toward the vacant O4 site, which makes incorporation energetically favorable (Supplementary Figure 4a). On the other hand, if W2 is incorporated into the center of the Mn1–(O5)–Mn4 bond, H_W2_^δ+^ is oriented toward cationic Mn1(III) and Mn4(III), causing repulsion (Supplementary Figure 4b). Indeed, QM/MM calculations show that neither W2 nor W3 was incorporated into the vacant O5 site of the O5-depleted Mn_4_CaO_4_ cluster. Depletion of O5 did not even affect the H-bond network near O5, including the short low-barrier H-bond between TyrZ and D1-His190 (~2.5 Å)

D1-Asp61, the only acidic residue that is not a ligand near the Mn_4_CaO_5_ cluster (i.e., a second sphere ligand), accepted an H-bond from W539, delocalizing the negative charge over [D1-Asp61…W539]^–^ and inducing O_W539_^δ–^. Thus, D1-Asp61 provides a driving force for the incorporation (i.e., it facilitates interactions between O_W539_^δ–^ and Mn3(III)/Mn4(III)) (Fig. 5). Indeed, it has been reported that O_2_ release was dramatically decelerated in the D1-D61N mutant^50–52^. As long as O_2_ was present in the Mn3/Mn4 moiety, H_2_O at W539 did not incorporate into the O4-binding site (Supplementary Figure 4), i.e., the presence of O_2_ at the Mn3/Mn4 moiety sterically inhibited incorporation of H_2_O at W539 into the O4 site. Therefore, the exergonic incorporation of W539, which was facilitated by D1-Asp61, could contribute to expulsion of O_2_ (Supplementary Figure 4).

**Fig. 6:**
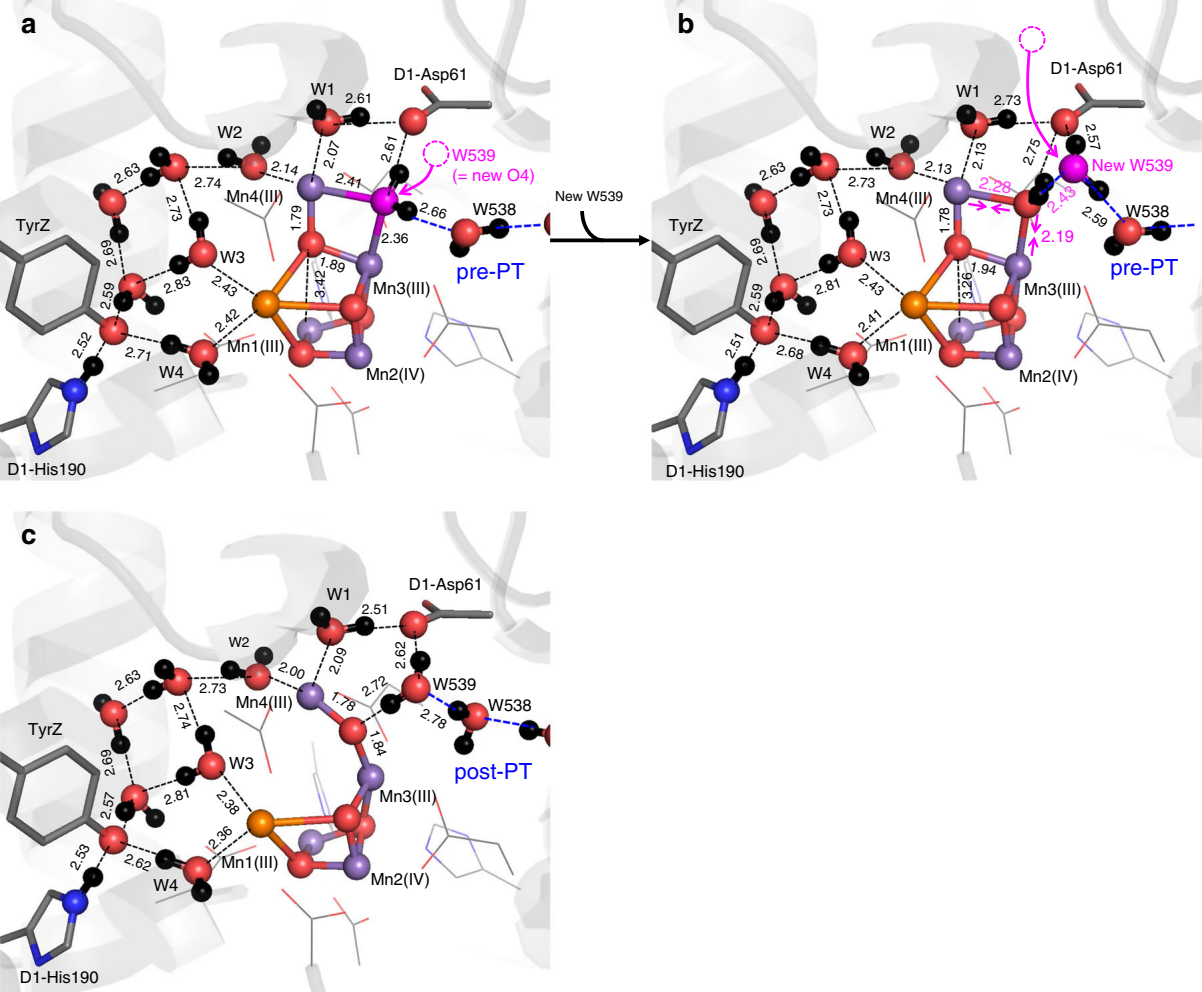
QM/MM geometries in the O4-depleted and O5-depleted PSII proteins. **a** Formation of the Mn3–O_W539_ and Mn4–O_W539_ bonds in response to incorporation of H_2_O at W539 into the O4 site of the O4-depleted Mn_4_CaO_4_ cluster. **b** Shorting the Mn3–O_W539_ and Mn4–O_W539_ bonds in response to incorporation of H_2_O into the vacant W539 site. **c** QM/MM geometries in the O5-depleted PSII. (Mn1, Mn2, Mn3, Mn4) = (III, IV, III, III)

W539 forms an H-bond with D1-Ser169 (O_W539_–O_D1-Ser169_ = 2.73 Å), which has been proposed to provide access to water molecules for the substrate-binding site^42^. Incorporation of W539 into the O4 site was also observed in QM/MM calculations using the original PSII crystal structure and depleting the O4 atom (Fig. 6a); the obtained geometry was consistent with the O4-incorporated geometry obtained through the S_2_, S_3_, and S_4_ states (Fig. 4), which indicates the robustness of the present reaction scheme.

As H_2_O at W539 was incorporated into the Mn_4_CaO_4_ cluster, the W539 site became vacant. When the W539 site was refilled by a water molecule, both Mn3–O_W539_ and Mn4–O_W539_ were further shortened to ~2.2 Å (Fig. 6b). It seems likely that incorporation of a water molecule via D1-Asp61 into the W539 site^16^ led to fixation of O4 as a component of the Mn_4_CaO_5_ cluster.

### Alteration of the H-bond pattern along the O4-water chain

QM/MM calculations indicated that O4 may exist as OH^–^ in S_0_, and release of the proton occurs in the S_0_-to-S_1_ transition along the O4-water chain (Fig. 1), with transforming of the pre-PT to a post-PT H-bond pattern^4^. This implies that the pre-PT pattern must be recovered from the post-PT pattern before the next turnover.

**Fig. 7:**
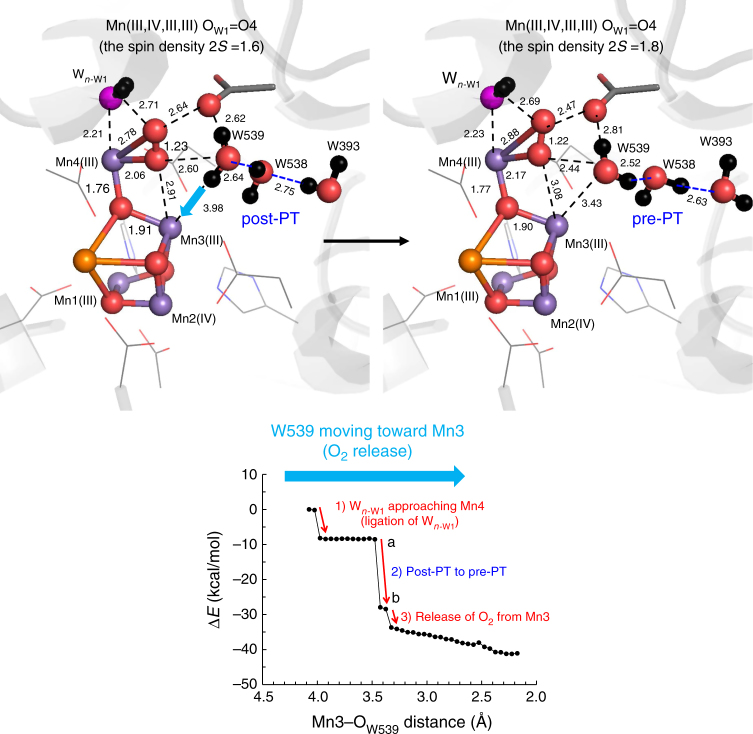
The energy profiles when W539 approaches Mn3(III). As W539 approaches Mn3(III) (i.e., the Mn3…O_W539_ distance was decreased), concertedly (1) W_*n*__-W1_ approaches Mn4 (ligating to Mn4), (2) the H-bond pattern of the O4-water chain transforms from the post-PT to pre-PT pattern, and (3) O_2_ releases from the Mn3 moiety. The total spin density 2*S* is shown for O_W1_=O4

As W539 approached Mn3 during O_2_ evolution, the H-bond pattern along the O4-water chain (e.g., W538) transformed from the post-PT pattern to the pre-PT pattern, since W539 (i.e., new O4) needs to reorient to form a μ-oxo bridge (Mn3–O_W539_–Mn4) (Fig. 7). Given that release of the first proton from H_2_O at O4 occurs via D1-Asp61 in the pre-S_0_-to-S_0_ transition (i.e., the O4-water chain is not used), the O4-water chain remains in the pre-PT pattern, ready for release of the proton in the S_0_-to-S_1_ transition.

The rate-limiting step in O=O formation has been reported to exhibit a *k*_D_/*k*_H_ of 1.4 ^6^, which is consistent with the *k*_D_/*k*_H_ value for transformation between pre- and post-PT patterns^11^. This may correspond to H-bond transformation along the O4-water chain (Fig. 7), since the corresponding water chain or H-bond network is absent at the O5/O6 moiety^9,17,18^.

### Characteristic O sites in the two-flash structure

The two-flash illuminated PSII structure shows the extremely short averaged O4…O_W539_ distance of 2.32 Å (2.47 and 2.17 Å)^17^. The present QM/MM calculations reproduced the short O4–O_W539_ distance (2.43 Å) when H_2_O at W539 was incorporated into the O4 site and the vacant W539 site was refilled by H_2_O (pre-S_0_ state, Fig. 6b). The absence of W538 (i.e., disordered W538) in the two-flash illuminated PSII structure^17^ can be explained by the present reaction scheme, as O4 is consumed and thus incorporation of W539 and displacement of W538 occur (Fig. 6). The vacant W539-binding site (Fig. 6a) can be refilled by H_2_O, as MD simulations indicated that bulk water could enter the W539 and W538 sites via D1-Asp61^16^. Even in the Mn-depleted PSII crystal structure, both W539 and W538 exist and are well-ordered (*B*-factor values are 33.9 and 43.4, respectively)^53^, highlighting the originally large binding affinity due to D1-Asp61. This, in turn, suggests that the absence of well-ordered W538 in the two-flash illuminated PSII structure^17^ is exceptional (Table 2), and can be understood only when reorganization of W539 and W538 occurs.

**Table 2:** *B*-factor values in the two-flash illuminated PSII structure (5WS6) and the 1.9-Å PSII structure (3ARC) (in Å^2^)

		*B*-factor			
		5WS6		3ARC	
Group	Atom	A monomer	B monomer	A monomer	B monomer
Mn_4_CaO_*x*_	CA1	46.90	58.02	24.57	27.49
	O1	48.53	74.66	22.48	25.07
	O2	43.90	50.86	25.22	27.28
	O3	46.58	53.58	23.27	27.14
	O4	50.67	50.33	26.62	27.74
	O5	55.69	57.35	24.13	28.14
	O6	56.68	54.48	—	—
	MN1	49.36	56.03	22.62	25.98
	MN2	49.76	54.85	22.88	26.69
	MN3	49.65	53.09	23.49	25.54
	MN4	54.02	56.60	26.26	28.67
D1-Val185	CB	42.42	44.24	20.76	22.11
	CG1	40.77	46.72	20.28	22.2
	CG2	43.17	42.46	19.93	22.27
W539^9^	O	54.55	56.75	23.89	25.27
(W567^17^)					
W538^9^	O	—	—	24.55	28.16
(W665^17)^					
W393^9^	O	49.14	46.52	24.15	24.36
W1	O	49.68	42.68	22.35	24.54

— not applicable

## Discussion

**Fig. 8:**
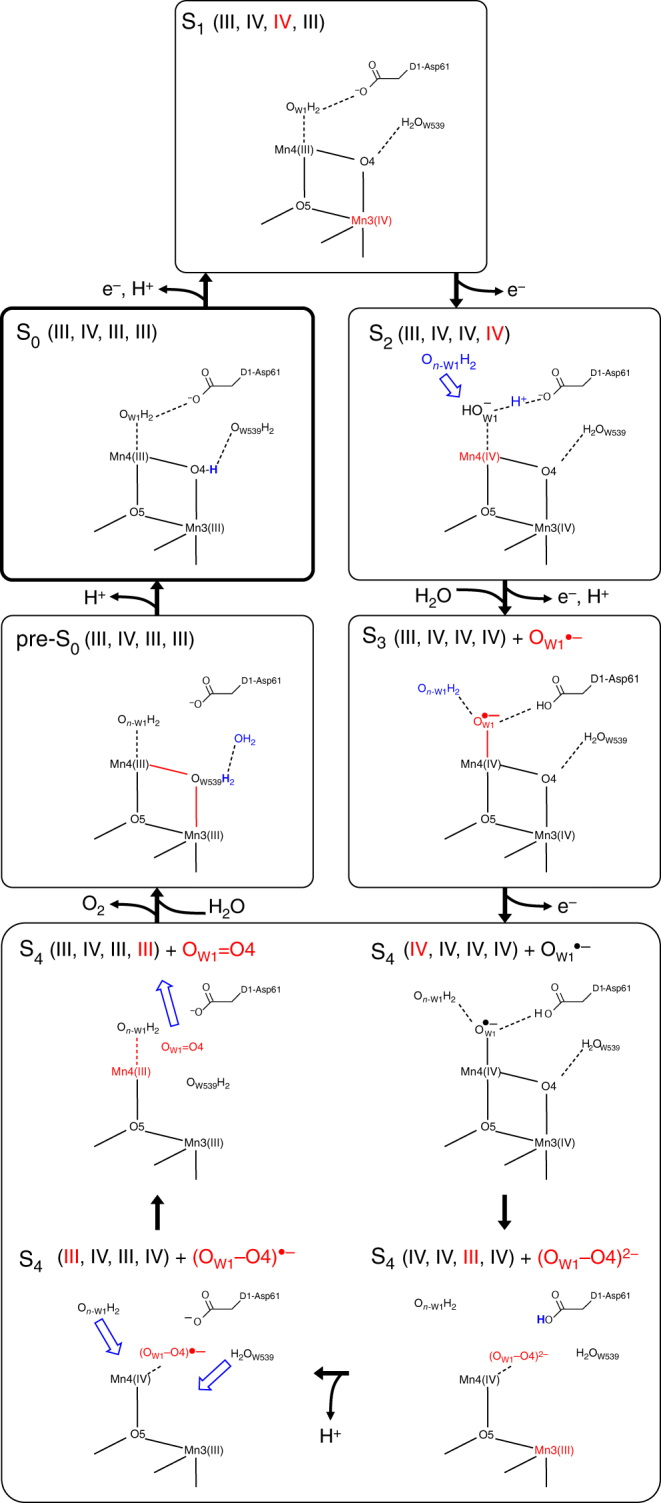
Mechanism of O_2_ formation and recovery of Mn_4_CaO_5_. S_0_ is the lowest oxidation state. In the S_0_-to-S_1_ transition, a low-barrier H-bond between OH^–^ at O4 and W539 (as indicated in the crystal structure^9^) releases the proton via the O4-water chain^4^. In the O4-water chain, the pre-PT pattern transforms into the post PT pattern as a result of proton transfer. In the S_1_-to-S_2_ transition, oxidation of Mn4(III) to Mn4(IV) occurs, resulting in a decrease in p*K*_a_ of W1 and formation of a low-barrier H-bond between W1 and D1-Asp61. In the S_2_-to-S_3_ transition, D1-Asp61 decreases the redox potential of W1 and facilitates proton-coupled oxidation of O_W1_H^–^ to O_W1_^•–^. O_W1_^•–^ attracts W_*n*__-W1_. In S_4_, oxo-oxyl radical coupling occurs between O_W1_^•–^ and corner μ-oxo O4, leading to formation of (O_W1_–O4)^2–^, reduction of Mn, incorporation of W_*n*__-W1_ as new W1, and approach of W539. In the S_4_-to-pre-S_0_ transition, as O_W1_=O4 moves away from the Mn_4_CaO_5_ moiety, water incorporation occurs near two second sphere ligands, D1-Asp61 and CP43-Arg357: D1-Asp61 accepts an H-bond from W539 and incorporation of W539 into the vacant O4 site near CP43-Arg357 leads to reorientation of W539 and formation of a μ-oxo bridge with Mn3–O_W539_ and Mn4–O_W539_. Reorientation of W539 (i.e., new O4) at the O4 site propagates along the O4-water chain, transforming the post-PT pattern to the pre-PT pattern. The vacant W539 site can be filled by a water molecule from the D1-Glu65/D2-Glu312 channel via D1-Asp61^16^. Pre-S_0_, where H_2_O is present at O4, can be stabilized by release of a proton from the newly incorporated O4 site and proceeds to S_0_

Based on the findings reported here, we are able to propose the following reaction scheme (Fig. 8). In S_2_, the H atom of H_2_O at W1 is significantly migrated toward D1-Asp61 in the open-cubane Mn(III,IV,IV,IV) (Fig. 2). The S_2_-to-S_3_ transition involves oxidation of O_W1_H^–^, resulting in formation of O_W1_^•–^ in Mn(III,IV,IV,IV), as suggested by Messinger et al.^32^. In S_3_, the binding of W1 at Mn4(IV) is pronounced due to formation of O_W1_^•–^. The binding of W_*n*__-W1_ at O_W1_^•–^ is also pronounced (Fig. 3). S_4_ is initially Mn(IV,IV,IV,IV) with O_W1_^•–^. Oxo-oxyl radical coupling between O4 and O_W1_^•–^ leads to formation of (O_W1_–O4)^2–^ and reduction to Mn(IV,IV,III,IV) (Fig. 4); W_*n*__-W1_ decreases the energy barrier for the (O_W1_–O4)^2–^ formation, serving as a catalyst. As O_W1_=O4 moves away from the Mn3/Mn4 moiety toward D1-Ile60 in the proposed O_2_-exiting pathway^48^, H_2_O at W539 is incorporated into the vacant O4 site, forming a μ-oxo bridge with Mn3–O_W539_ and Mn4–O_W539_ in a concerted exergonic process. W_*n*__-W1_ is also incorporated into the Mn_4_CaO_5_ cluster as the new W1 ligand. The two Mn3–O_W539_ and Mn4–O_W539_ bonds are further shortened when the vacant W539 site is refilled by a water molecule, which approaches via D1-Asp61^16^, recovering the original Mn_4_CaO_5_ structure. Incorporation of W539 into the O4 site also seems to lead to transformation of the H-bond pattern along the O4-water chain to the pre-PT pattern (Fig. 7), in readiness for proton transfer in the S_0_-to-S_1_ transition^4^.

The proton releasing sites are identified as W1 in the S_2_-to-S_3_ and the S_3_-to-S_0_ transitions and O4 in the pre-S_0_-to-S_0_ and S_0_-to-S_1_ ^4^ transitions. Based on these observations, W1 and O4 are deprotonatable substrate water molecules on the current turnover, and W_*n*__-W1_ and W539 serve as “holding sites^7^” and become W1 and O4, respectively, on the next turnover. Then, the D1-Glu65/D2-Glu312 channel serves as a water intake channel for both substrate water molecules^16^. The location of O4/W539 at the dead end of the narrow region of the D1-Glu65/D2-Glu312 channel^16^ may also make the exchange rate slow.

If the fast-exchanging and slow-exchanging water molecules represent two substrate water molecules, they might be W1 and O4, respectively. Then, the decrease in the exchange rate for the fast-exchanging water molecule in the S_2_-to-S_3_ transition^12^ might be explained by the pronounced binding of W1 at Mn4 (O_W1_^•–^ in S_3_) (Supplementary Figure 5). However, as suggested by Noguchi and coworkers based on FTIR difference spectroscopy, it seems also possible that the exchanging water molecule is not necessarily a substrate water molecules on the current turnover (W1 and O4 in the present reaction scheme) but rather a substrate water molecule on the next turnover (W_*n*__-W1_ as next W1 and W539 as next O4)^41^. In this case, the increase in the exchange rate of the slow-exchanging water molecule (i.e., W539) in the S_1_-to-S_2_ transition^12^ is due to the relaxed H-bond network of the O4-water chain (including O4 and W539) in the S_1_-to-S_2_ transition (i.e., after proton transfer) with respect to the strongly coupled H-bond network in the S_0_-to-S_1_ transition^4^. The decrease in the exchange rate for the fast-exchanging water molecule (i.e., W_*n*__-W1_) in the S_2_-to-S_3_ transition^12^ can be explained by the pronounced binding of W_*n*__-W1_ at O_W1_^•–^.

W_*n*__-W1_, a substrate on the next turnover, decreases the energy barrier for (O_W1_–O4)^2–^ formation in S_4_, serving as a catalyst. W539, another substrate on the next turnover, is the direct proton acceptor for the current turnover substrate H_2_O at O4. The remarkable cooperativity of the next substrate water molecules to catalyze the current substrate water molecule (Fig. 4b), as well as the self-recovery of the Mn_4_CaO_5_ cluster from the O_2_-evolved Mn_4_CaO_4_ cluster in a concerted exergonic process (Fig. 4a), suggest that W1/ W_*n*__-W1_ and O4/W539 are substrate water molecules.

## Methods

### Coordinates and atomic partial charges

The atomic coordinates of PSII were taken from the X-ray structure of PSII monomer unit “A” of the PSII complexes from *Thermosynechococcus vulcanus* at a resolution of 1.9 Å (PDB code, 3ARC)^9^. During optimization of hydrogen atom positions with CHARMM^54^, the positions of all heavy atoms were fixed and all titratable groups (e.g., acidic and basic groups) were ionized. In QM/MM calculations, additional counter ions were added to neutralize the entire system. Atomic partial charges of the amino acids and cofactors were obtained from the CHARMM22^55^ parameter set and our previous studies on PSII^4^, respectively. D1-His337 was considered to be protonated^56^.

### QM/MM calculations

The unrestricted density functional theory (DFT) method was employed with the B3LYP functional and LACVP* basis sets, using the QSite^57^ program. In the QM region, all the atomic coordinates were fully relaxed. In the MM region, the positions of H atoms were optimized using the OPLS2005 force field, while the positions of heavy atoms were fixed. The cluster was considered to be in the S_2_, S_3_, S_4_, and S_0_ states with antiferromagnetically coupled Mn ions; the resulting Mn oxidation states (Mn1, Mn2, Mn3, Mn4) and the total spin *S* were as follows: (III, IV, IV, IV) and *S* = 7/2 (↑↓↑↑) in S_2_; (III, IV, IV, IV) + O_W1_^•–^ and *S* = 8/2 (↑↓↑↑ + ↑) in S_3_; (IV, IV, IV, IV) + O_W1_^•–^ and *S* = 7/2 (↑↓↑↑ + ↑), (IV, IV, III, IV) + (O_W1_–O4)^2–^ and *S* = 7/2 (↑↓↑↑), and (III, IV, III, III) + O_W1_=O4 and (↑↓↑↑ + ↓) in S_4_; (III, IV, III, III) and *S* = 9/2 (↑↓↑↑) in pre-S_0_ (Table 1, Supplementary Figures 1 and 3 for other spin configurations). The Mn_4_CaO_5_ geometry in S_*n*_ was obtained as follows: first we prepared the QM/MM-optimized S_*n*_ geometry with ferromagnetically coupled Mn ions, using the QM/MM-optimized S_*n*__–1_ geometry. The resulting S_*n*_ geometry was then optimized as antiferromagnetically coupled Mn ions. The initial-guess wave functions were obtained by using the ligand-field theory^58^ implemented in the QSite program. See Supplementary Data 1 for the atomic coordinates.

To analyze S_2_, S_3_ (Fig. 2), and S_4_ (Fig. 4), the QM region (small QM) was defined as the Mn_4_CaO_5_ cluster (including the ligand side-chains of D1-Asp170, D1-Glu189, D1-His332, D1-Glu333, D1-Asp342, and CP43-Glu354, and the backbone of D1-Ala344); the ligand water molecules of W1-W4; the O4-water chain (W539, W538, and W393); the W_*n*__-W1_-binding site (W_*n*__-W1_, W426, and W614); the Cl-1-binding site (Cl-1, W442, W446, and side-chains of D1-Asn181 and D2-Lys317); and the second sphere ligands (side-chains of D1-Asp61 and CP43-Arg357). Other groups were approximated by the MM force field. To mainly analyze the O4-depleted and O5-depleted PSII structures in S_0_ (Fig. 6), the QM region (large QM) was defined as the Mn_4_CaO_5_ cluster (including the ligand side-chains of D1-Asp170, D1-Glu189, D1-His332, D1-Glu333, D1-Asp342, and CP43-Glu354, and the backbone of D1-Ala344); the ligand water molecules of W1-W4; the O4-water chain (W539); the Cl-1-binding site (Cl-1, W442, W446, and side-chains of D1-Asn181 and D2-Lys317); the second sphere ligands (side-chains of D1-Asp61 and CP43-Arg357); the H-bond network of TyrZ (side-chains of D1-Tyr161, D1-His190, and D1-Asn298), and the diamond-shaped cluster of water molecules^59^ (W5, W6, and W7).

To obtain the potential-energy profiles of H-bonds (Fig. 2b and Supplementary Fig. 1), the QM/MM optimized geometry was used as the initial geometry. The H atom under investigation was moved from the H-bond donor atom (O_donor_) toward the acceptor atom (O_acceptor_) by 0.05 Å, after which the geometry was optimized by constraining the O_donor_–H and H–O_acceptor_ distances and the energy was calculated. This procedure was repeated until the H atom reached the O_acceptor_ atom. To obtain the potential-energy profiles of (O_W1_–O4)^2–^ formation (Fig. 4b and Supplementary Fig. 3) and O_2_ release (Fig. 7 and Supplementary Fig. 4), the QM/MM optimized geometry was used as the initial geometry; e.g., the O_W1_…O4 distance was then decreased by 0.05 Å, after which the geometry was optimized by constraining the O_W1_…O4 distance and the energy was calculated. This procedure was repeated until formation of (O_W1_–O4)^2–^.

### MD simulations

The PSII structure was embedded in a lipid bilayer, which is composed of 546 1-palmitoyl-2-oleyl-sn-glycero-3-phosphocholine (POPC), and soaked in 78889 flexible water molecules (SPC-Fw)^60^, using the CHARMM-GUI program^61^. After geometry optimization with position restraints on heavy atoms, the system was heated from 0.001 K to 300 K during 5.0 ps. The position restraints on heavy atoms were gradually released over 16.5 ns. An equilibrating MD run was conducted for 45 ns using the MD engine AMBER 14^62^ with the SHAKE algorithm for hydrogen constraint^63^. The production run was conducted with an MD time step of 0.5 fs without hydrogen constraint, using AMBER 14. To control temperature and pressure, the Berendsen thermostat and barostat were employed ^65^. See ref. ^16^ for force field parameters.

### Data availability

All the data supporting the findings of this study are available within the article and its Supplementary Information files or from the corresponding author upon reasonable request.

## Electronic supplementary material

Supplementary Information

Description of Additional Supplementary Files

Supplementary Data 1
