# Supplementary material for: O2 evolution and recovery of the water-oxidizing enzyme
Source: Nat Commun. 2018 Mar 28;9:1247. doi: 10.1038/s41467-018-03545-w (PMC5871790; doi:10.1038/s41467-018-03545-w)
Supplement: Supplementary file 1 — Supplementary Information [file 41467_2018_3545_MOESM1_ESM.docx]

**Supplementary Information**

O_2_ evolution and recovery of the water-oxidizing enzyme

Kawashima et al.

**Supplementary Figures**

**Supplementary Figure 1.** The open-cubane S_2_-to-S_3_ transition. (a) QM/MM geometries in the open-cubane S_2_-to-S_3_ transition in the ferromagnetically coupled form (*S* = 13/2 in S_2_ and 12/2 in S_3_). (b) The energy profiles of the H-bond between W1 (H_2_O/OH^–^ in S_2_, i.e., the first released proton, and OH^●^/O^●–^ in S_3_, i.e. the second released proton) and D1-Asp61. The black curve corresponds to release of the proton from H_2_O at W1 to D1-Asp61 in S_2_, whereas the red curve corresponds to release of the proton from OH^●^ at W1 to D1-Asp61 in S_3_.

**Supplementary Figure 2.** Reorientation of the D1-Asp61 side-chain in MD simulations. A typical H-bond pattern (a) before 34 ns (i.e. before reorientation) and (b) after 37 ns (i.e., reorientation). (c) Distribution of the protonated D1-Asp61 side-chain before 34 ns and (d) after 37 ns (10 snapshots). Note that at 34−37 ns, W1 donates an H-bond to the protonated O site of D1-Asp61 (i.e., without re-orientating the D1-Asp61 side chain). Dotted lines indicate H-bonds.

**Supplementary Figure 3.** The S_4_ and pre-S_0_ states. (a) QM/MM geometries in the ferromagnetically coupled high spin form (*S* = 13/2 in S_4_ and *S* = 15/2 in pre-S_0_). Mn, Ca, O, and H atoms are represented by purple, orange, red, and black balls, respectively. Dotted lines indicate distances (excluding H atoms). (b) The energy profiles of the (O_W1_–O4)^2–^ formation (the first and second panels in (a)) in the presence (blue curve) and absence (red curve) of W_n-W1_. The initial lower barriers correspond to reorientation of the carboxylate group of D1-Asp61 with respect to O_W1_^●–^/(O_W1_–O4)^2–^. In all S-state transitions, including the (O_W1_–O4)^2–^ formation process, Mn2(IV) is not involved in oxidation/reduction, which could explain the similar energy barriers in the antiferromagnetically (↑↓↑↑) (Figure 4) and ferromagnetically (↑↑↑↑) (Supplementary Figure 3) coupled forms.

**Supplementary Figure 4.** The energy profiles for release of (O_W1_–O4) ^●–^. (O_W1_–O4) ^●–^ moves away from the Mn3 moiety. As (O_W1_–O4) ^●–^ moves away from the Mn3 moiety, concertedly 1) W_n-W1_ approaches Mn4 (ligating to Mn4) and O_W1_=O4 forms, and 2) W539 is incorporated into the O4 site.

**Supplementary Figure 5.** Possible mechanism for water exchange. In case the fast-exchanging and slow-exchanging water molecules represent two substrate water molecules. Red and blue arrows indicate fast-exchanging and slow-exchanging water molecules, respectively. Dotted blue arrows indicate very slow exchange processes. Note that W1 is exchangeable with bulk water via the D1-Glu65/D2-Glu312 channel (red double-headed arrows). If the fast-exchanging and slow-exchanging water molecules represent two substrate water molecules, they might be W1 and O4, respectively. In S_2_ and S_3_, the exchange rate of the slow-exchanging water molecule increases 100 times with respect to S_1_ [^1^](#_ENREF_1). O^2–^ at the O4 site might possibly be more exchangeable with charged OH_W1_^–^ (S_2_) and O_W1_^●–^ (S_3_) than with uncharged H_2_O_W1_ (S_1_) (note, a similar mechanism is proposed for W2 and O4 in ref. [^2^](#_ENREF_2)).

**Supplementary Tables**

**Supplementary Table 1.** Occupancies (0 = unoccupied and 1 = fully occupied) of the W_n-W1_ water molecule in S_2_ and S_3_ in MD trajectories (1 to 6).

| trajectory | occupancy (W_n-W1_) | | |
| --- | --- | --- | --- |
|  | S_2_ | S_3_ | ratio (= S_3_/S_2_) |
| 1 | 0.006 | 0.352 | 63 |
| 2 | 0.013 | 0.314 | 25 |
| 3 | 0.028 | 0.154 | 5 |
| 4 | 0.019 | 0.128 | 7 |
| 5 | 0.008 | 0.183 | 23 |
| 6 | 0.025 | 0.373 | 15 |
|  |  |  |  |
| average | 0.016 | 0.251 | 15 |

**Supplementary References.**

1 Hillier, W. & Wydrzynski, T. The affinities for the two substrate water binding sites in the O_2_ evolving complex of photosystem II vary independently during S-state turnover. *Biochemistry* **39**, 4399-4405 (2000).

2 Vinyard, D. J. & Brudvig, G. W. Progress Toward a Molecular Mechanism of Water Oxidation in Photosystem II. *Annual Review of Physical Chemistry* **68**, 101-116 (2017).
