# Supplementary material for: O2 evolution and recovery of the water-oxidizing enzyme
Source: Nat Commun. 2018 Mar 28;9:1247. doi: 10.1038/s41467-018-03545-w (PMC5871790; doi:10.1038/s41467-018-03545-w)
Supplement: Supplementary file 2 — Description of Additional Supplementary Files [file 41467_2018_3545_MOESM2_ESM.docx]

**Description of Additional Supplementary Files**

File Name: Supplementary Data 1

Description: QM/MM-optimized geometries.
